# Value of ECV quantification in PAH: correlation with echocardiographic, MRI and serum biomarkers

**DOI:** 10.1186/1532-429X-17-S1-P295

**Published:** 2015-02-03

**Authors:** Sandrine Hubert, Gilbert Habib, Alexis Jacquier

**Affiliations:** 1 Radiologie, CHU la Timone, Marseille, France; 2 Cardiologie, Assistance publique hopitaux de Marseille, Marseille, France

## Background

The evaluation of right ventricle (RV) function is paramount in pulmonary hypertension (PAH) although it can be difficult to assess. Right ventricular myocardial fibrosis assessed using late gadolinium enhancement (LGE) has been correlated with right ventricular function and prognosis. Myocardial fibrosis can be evaluated by several methods, including LGE, ECV quantification using native and post contrast T1 mapping sequence and serum markers of collagen turnover. Value of ECV quantification and it's correlation with other biomarkers has not been systematically assessed during PAH. The goal of that study is to compare ECV, echo longitudinal strain, and serum markers of collagen turnover in the assessment of myocardial fibrosis in a population of adults with PAH.

## Methods

Twelve patients with PAH (6 male) were prospectively included and underwent the same day:

- Trans thoracic echocardiography: including measurement of right ventricular fractional shortening (RVFS) (%)by 2D echo, TAPSE by M-mode (mm), maximal tricuspid annulus velocity S (m/s) by tissue Doppler, RV global deformation RVD (%) and segmental (septal and lateral) RV deformations (%) by 2D strain

- 1.5T CMR including 1) right ventricular volumes and ejection fraction using standard SSFP sequence 2) native, and 10 to 15 min post contrast (0.2mmol/kg; DOTAREM) T1 mapping using Modified MOLLI sequence (4-3-2, 3 heart beats between acquisition)

- Serum quantification of 2 biomarkers of collagen turnover: TIMP-1 (Tissue Inhibitor of MetalloProteinase 1) and PIII-NP (Procollagen III N-Propeptid), and hematocrit.

## Results

Global RV ECV, reflecting the quantity of myocardial fibrosis, was abnormal (> 25%) for our entire population. We found a significant correlation between global RV ECV and four prognostic parameters for PAH: RV fractional shortening (RVOTfs) (r=0.6, p=0.026) S' wave velocity (r=0.7, p=0.009), TAPSE (r=0.6, p=0.040) and RV systolic function on the MRI (r=0.6, p=0.04). There was a correlation between the ECV values for the lateral wall of the RV and the ECV of the septum (r= 0.4, p=0.206). We also found a significant correlation between septal ECV and 2D septal strain (r=0.7, p=0.013). Lastly, we recorded a trend suggesting significance between the PIIINP values and global ECV in the sub-group 7 patients (p= 0.064).

## Conclusions

ECV quantification in PAH appears to correlate with known prognostic biomarkers and more specifically with the biomarkers that assess RV systolic function. These results suggest that RV myocardial fibrosis could become a new prognostic parameter in PH.

